# The effectiveness of a Kampo e-learning course incorporated into the medical education curriculum: a possible solution to instructor shortages and time constraints

**DOI:** 10.1186/s12909-025-06874-9

**Published:** 2025-02-24

**Authors:** Yoshinobu Nakada, Makoto Arai, Ippei Yamato, Tatsuya Nogami, Hiroshi Odaguchi, Daigo Taniguchi, Jun Tomita, Tomoaki Ishigami

**Affiliations:** 1 Department of Kampo Medicine, Tokai University School of Medicine, 143 Shimokasuya, Isehara, Kanagawa 259-1193 Japan; 2 Center for Oriental Medicine, Shonan Hospital, 1-1-1 Takatori, Kanagawa 237-0067 Yokosuka, Japan; 3 Department of General Internal Medicine, St. Marianna University School of Medicine, 2-16-1 Sugao, Miyamae-ku, Kanagawa 216-8511 Kawasaki, Japan; 4 Department of Medical Education, Tokai University School of Medicine, 143 Shimokasuya, Isehara, Kanagawa 259-1193 Japan; 5 Oriental Medicine Research Center, School of Pharmacy, Kitasato University, 5-9-1 Shirokane, Tokyo 108-8641 Minato-ku, Japan; 6 Yokohama City University School of Medicine, 3-9, Fukuura, Kanazawa-ku, Kanagawa 236-0004 Yokohama, Japan; 7 Department of Cardiology, Yokohama City University Hospital, 3-9, Fukuura, Kanazawa-ku, Kanagawa 236-0004 Yokohama, Japan

**Keywords:** E-learning, Kampo medicine, Effectiveness survey, Instructor shortages

## Abstract

**Background:**

The shortage of instructors and time to teach traditional Japanese (Kampo) medicine (KM) limits students’ understanding of its usefulness. We developed an e-learning course to solve this problem.

**Methods:**

The Kampo e-learning course consists of 12 lessons on 10 essential Kampo formulas with related formulas, proper prescriptions, dosages, and adverse reactions, followed by review questions. After the course, and each student answers 10 additional clinical questions correctly, they are awarded a certificate of completion. This e-learning course was first taught in 2022. The students were informed before taking the course that points would be added to their final test scores with proof they completed the e-learning course. A total of 119 third-year Tokai University School of Medicine students participated. To evaluate the effectiveness of the e-learning course, they were each asked 5 clinical questions, randomly selected from 10 pooled questions, then 4 awareness questions on their interest in, the necessity for, and their understanding of KM, and their perceived usefulness of the course before and after taking it. Wilcoxon’s signed-rank test was used to compare the changes.

**Results:**

The students’ pre- and post-course test scores were compared. The evaluations of “Interested in KM” and “Necessity of KM for clinical routines” and “Understanding how to use KM” improved significantly; however, “Usefulness of e-learning for studying Kampo medicine” did not change. Clinical question scores improved significantly.

**Conclusions:**

All the students completed the course resulting in significantly higher scores, proving this course’s effectiveness. Developed not only for students but also for novices, this new Kampo e-learning course can be incorporated into regular curriculums and made an easily accessible tool in clinical settings.

**Trial registration:**

This study is not a clinical trial but a kind of questionnaire survey, so that clinical trial number is not applicable.

**Supplementary Information:**

The online version contains supplementary material available at 10.1186/s12909-025-06874-9.

## Background

Japanese traditional (Kampo) medicine (KM) was introduced from China centuries ago and uniquely developed in Japan during the Edo Period (1603–1868) [1, 2]. KM, which was the primary medicine in Japan until the end of the Edo period, was taken over by Western medicine during the Meiji period (1868–1912).

Owing to KM being included in the Japanese National Health Insurance program in 1967, nowadays, 70–90% of Japanese physicians regularly prescribe KM in their medical practices according to clinical evidence and reports of mechanisms of action and/or by following guidelines from modern Western medicine [3–5]. However, not many physicians prescribe KM according to proper Kampo theory, which is essential to provide patients with the necessary individualized prescriptions, dosages, and therapy.

On the other hand, although Kampo theory differs from Western medical theory, prescribing KM has been approved if the physician has a valid Japanese medical license. However, questions on the Japanese medical licensing exam are mainly based on Western medicine. Therefore, medical students mostly study Western medical concepts to prepare for the exam. Moreover, although KM now is taught in all 82 medical schools in Japan, sufficient lecture time to understand Kampo theory correctly is not given resulting in the unavailability of enough qualified Kampo instructors. This lack of qualified instructors and insufficient lecture time are the two largest obstacles in Kampo education [6, 7]. Furthermore, Kampo education for medical residents and novice physicians share similar conditions [8]. Presumably, because of their busy schedules, physicians need learning tools with concise explanations that they can access easily, anytime and anywhere. In Japan, Kampo medicine has recently become a required course not only in medical but also in dental, pharmaceutical, and nursing model core curriculums. Kampo medicine is widely taught in all of these fields [9], albeit inadequately because of the reasons previously given. Common knowledge of Kampo medicine is supposed to be provided for people in these fields so that they have an equal and common understanding of its use. To facilitate such a goal, this new e-learning course could be used for all medical students and personnel worldwide.

These types of e-learning lessons, which have emerged in the digital era and flourished during the Covid-19 pandemic, are quite useful to students and novices to easily access information using their personal computers, tablets, and even smartphones, anytime and anywhere. Furthermore, e-learning lessons can easily be incorporated into regular lectures and active learning situations [10] and could be useful to physicians for referencing during clinical routines. This could be a solution to the lack of qualified Kampo instructors and the insufficient lecture time. Full-time instructors can deliver interactive lectures that cultivate trust, subject to student engagement. In contrast, e-learning operates in a unidirectional manner. Nonetheless, it provides students with the opportunity to freely revisit and review content they may have overlooked or wish to reinforce. As shown in the 2011 and 2019 survey comparison paper, the average duration of a Kampo medicine class was 8.28 sessions (about 60 min per session, so the required time is 500 min) [6, 7], while the time required for this e-learning course is approximately 160 min, which is shorter. However, since the time required for Kampo medicine lectures tends to be shortened due to the reform of medical education in Japan, this e-learning course could be applied as a tool for students to prepare for classes. Thus, e-learning lessons could be especially advantageous in Kampo education, in which those problems presently remain unsolved.

We therefore developed a new Kampo e-learning course for students and novices to improve their understanding and evaluated its effectiveness.

## Methods

The Kampo e-learning course was created and implemented by the Kanagawa 4 University Schools of Medicine Faculty Development Forum: Tokai University, Kitasato University, Yokohama City University, and St. Marianna University School of Medicine.

According to discussions with Kampo specialists, we initially chose the 10 most commonly used Kampo formulas that students and novices must know how to use. These formulas were selected taking into account Kampo theory, i.e., “Yin and yang,” “Deficiency and excess,” “Cold and heat,” “Exterior and interior (location),” " The six-stage pattern,” “Qi, blood, and fluid,” “The five viscera,” and others.

**Fig. 1:**
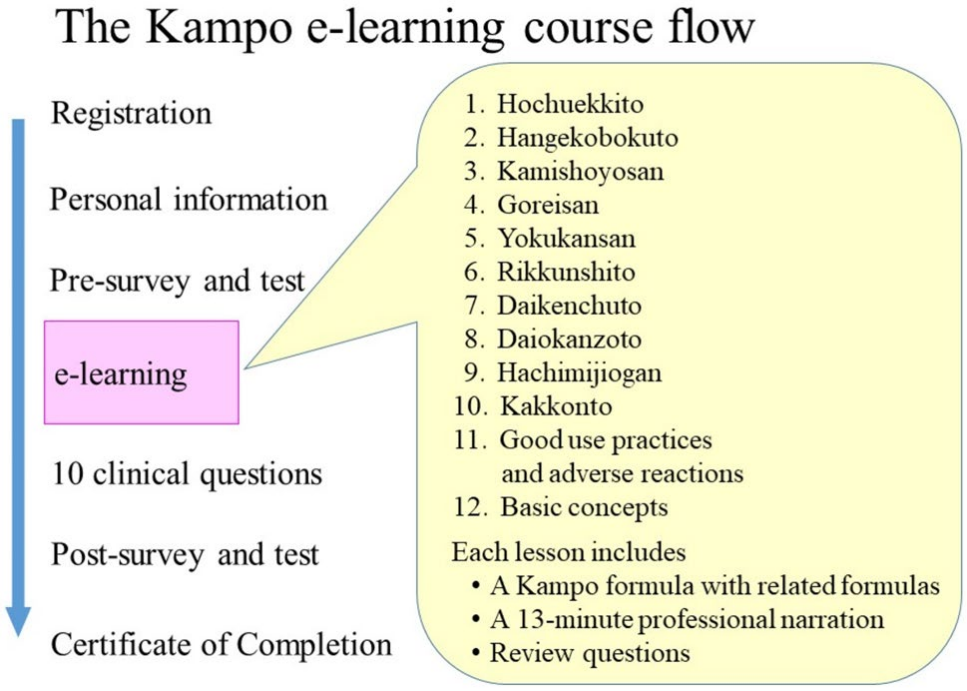
The Kampo e-learning course flow After registration and personal information has been entered, the pre-survey and test are given. The course consists of 12 lessons on 10 essential Kampo formulas with related formulas, prescription guidelines, adverse reactions, history, characteristics, etc. If the participants answer the 10 clinical questions correctly, the post-survey and test scores are given, and a certificate of completion is awarded

The Kampo e-learning course consists of 12 lessons (Fig. 1), which contain information on the 10 essential Kampo formulas with one lesson on “Good use practices and adverse reactions” and another on “Basic concepts.” The course can be easily accessed on personal computers, tablets, and smartphones. The lessons on the 10 essential Kampo formulas include related formulas, and prescription guidelines, Kampo theory, symptoms (to determine differential diagnoses), clinical evidence (physical exams, labs, and tests), differential prescriptions, and case presentations on: “Hochuekkito”, “Hangekobokuto”, “Kamishoyosan”, “Goreisan”, “Yokukansan”, “Rikkunshito”, “Daikenchuto”, “Daiokanzoto”, “Hachimijiogan”, and “Kakkonto” (Fig. 1). Each lesson is approximately 13 min long with professionally recorded narrations followed by review questions. After a student completes all 12 lessons and correctly answers the 10 additional clinical questions, a certificate of completion is awarded.

This e-learning course was assigned as homework in 2022 to be completed sometime from September 15 to 29. To decrease selection bias, third-year students who were generally novices in KM were selected. Furthermore, to increase the response rate, the students were told before the course that points would be added to their regular test scores when they showed their certificate of completion. All the 119 third-year Tokai University Medical School students participated in the course. Kampo education is integrated into the third-year curriculum, and this e-learning course was implemented as a component of the required Kampo medicine classes. To equalize the difficulty, each student was asked 5 clinical questions randomly selected from 10 pooled questions to objectively evaluate their knowledge acquisition by completing the course (Supplementary material). These questions encompassed nearly all of the 10 Kampo formulas, along with fundamental Kampo theory, including eight-principle syndrome differentiation, *qi*, *blood*, *fluid*, six-stage pattern, and five viscera. The students were not provided with the correct answers even after the course concluded, as these five questions were solely intended for the evaluation of the e-learning course. The scores before and after the course for these 5 clinical questions were compared. Then 4 awareness questions were asked to determine their subjective evaluation regarding “Interest in KM”, “Necessity for KM”, and “Understanding of KM”, and their “Perceived usefulness of the e-learning course” (Table 1). The scores for the 4 awareness questions were assigned as: “Very.” 3 points, “Slightly.” 2 points, “Hardly.” 1 point, and “Not.” 0 points. The questionnaire content is a 4-point scale based on the VRS (verbal rating scale). The questions were developed with reference to papers that have already been published [8]. The scores for each question were obtained from all the students before and after the course and statistically analyzed to evaluate its effectiveness.

**Table 1:** Questionnaire about KM

Same questions were asked before and after the Kampo e-learning course.	
1) Are you interested in KM?	
1. Very	
2. Slightly	
3. Hardly	
4. Not at all	
2) Do you think KM is necessary for clinical routines?	
1. Very	
2. Slightly	
3. Hardly	
4. Not at all	
3) Do you understand how to use KM?	
1. Very	
2. Slightly	
3. Hardly	
4. Not at all	
4) Do you think Kampo e-learning is useful for studying KM?	
1. Very	
2. Slightly	
3. Hardly	
4. Not at all	

This survey was approved (# 21R-039) by the Institutional Review Board for Clinical Research of Tokai University School of Medicine and conformed to the principles of the Helsinki Declaration. To improve Kampo education, informed consent was obtained to use the participants’ personal information regarding their careers, learning level of Kampo medicine, and pre- and post-course test scores. The participants were guaranteed that their names and e-mail addresses would remain confidential.

Wilcoxon’s signed-rank test was used to compare the changes before and after the course for statistical analyses.

## Results

**Fig. 2:**
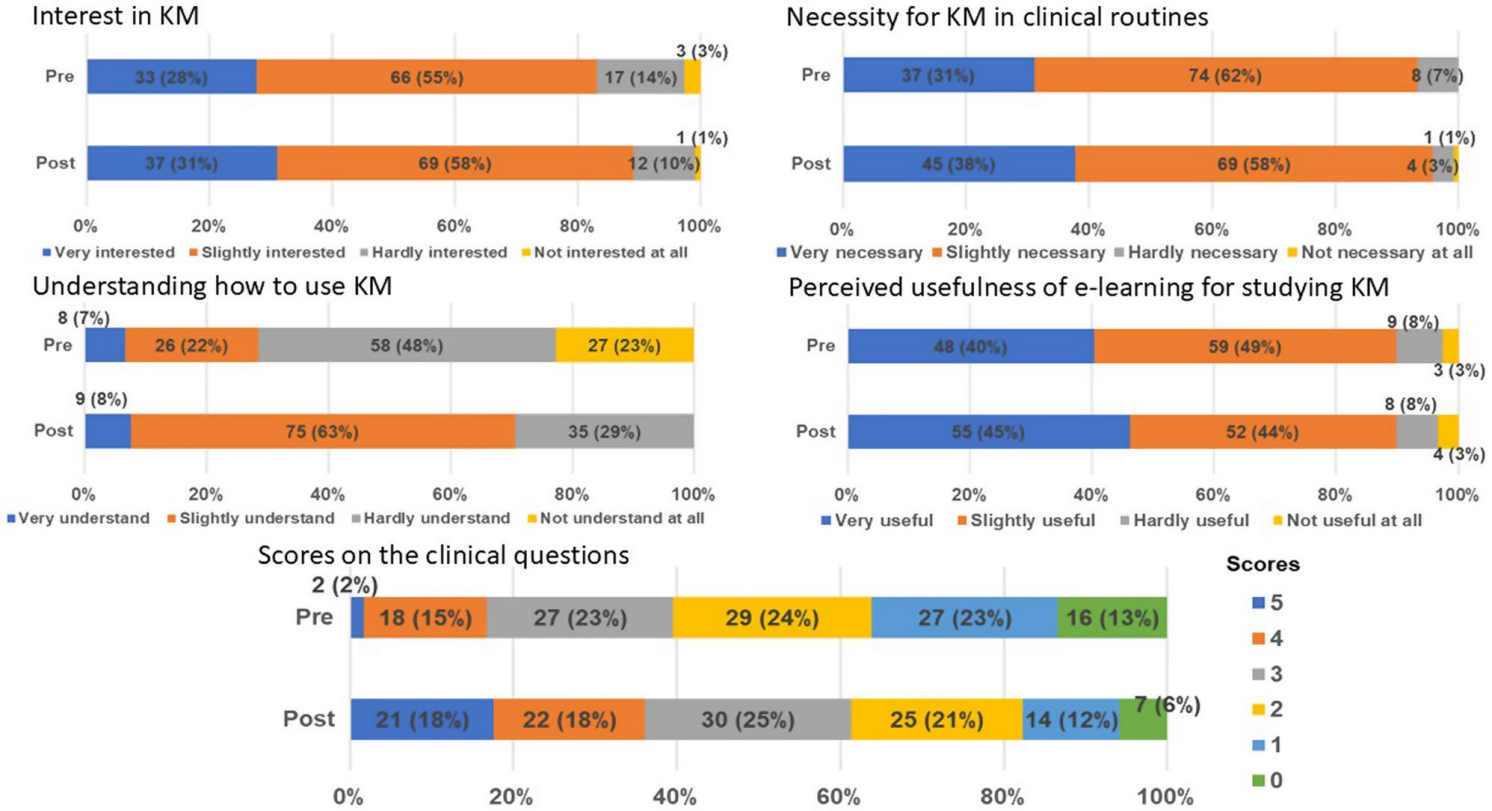
Comparison of 5 clinical and 4 awareness questions pre- and post-course to evaluate the e-learning course’s effectiveness

After the course, “Interested in KM” and “Necessity of KM for clinical routines” changed from 83% and 93% to 89% and 96%, respectively. Moreover, “Understanding how to use KM” dramatically increased from 29 to 71%. “Usefulness of e-learning for studying KM” was unchanged. The scores on the clinical questions (5-point Likert scale) improved (Fig. 2).

**Fig. 3:**
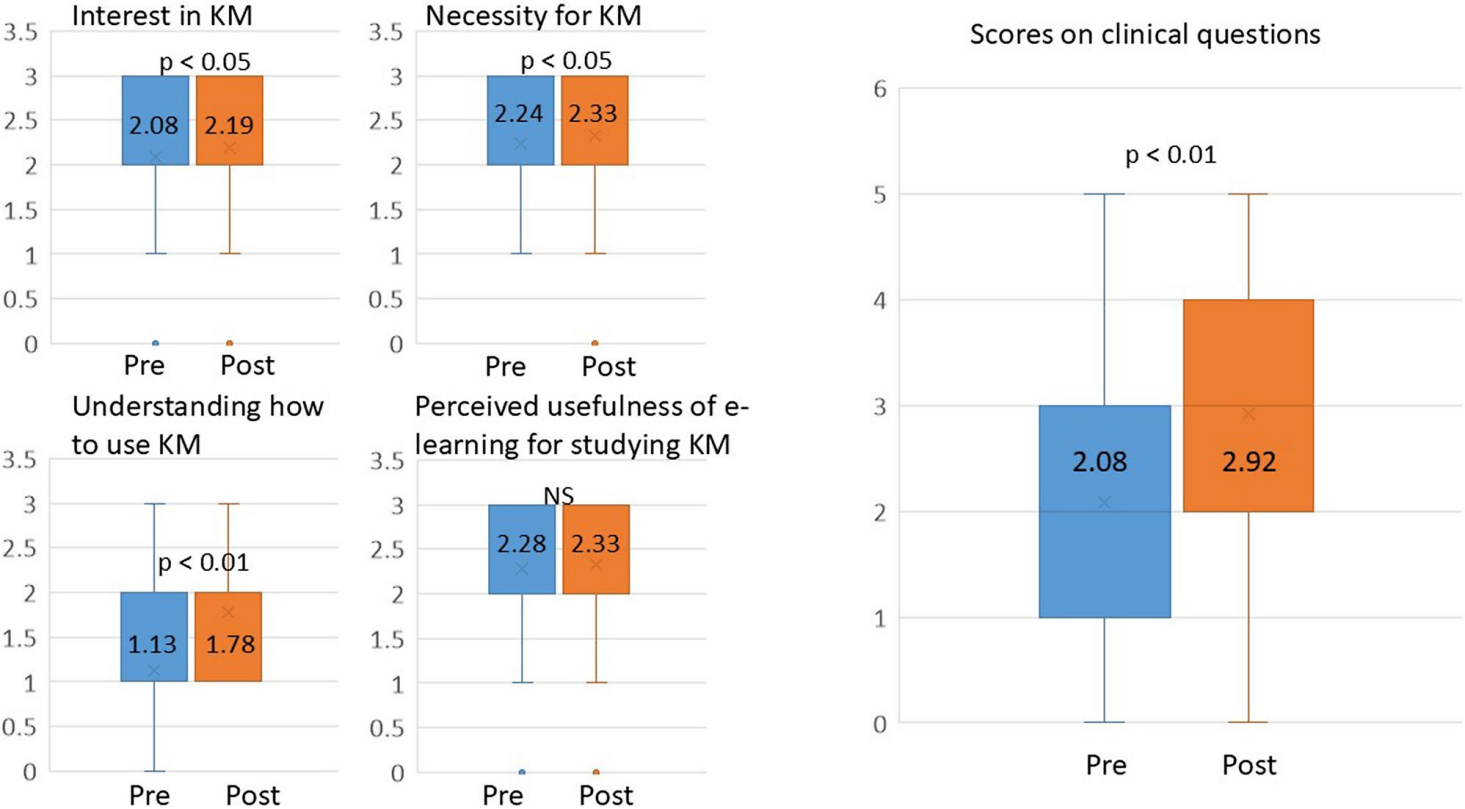
Statistical analyses of 5 clinical and 4 awareness questions pre- and post-course to evaluate the e-learning course’s effectiveness. Wilcoxon’s signed-rank test was used to compare the changes. The average values ​​are plotted on the graph

The scores on the 4 awareness questions were evaluated statistically. The scores for “Interest in KM,” “Necessity for KM,” and “Understanding of KM” increased significantly from 2.08 to 2.19 (*p* < 0.05), from 2.24 to 2.33 (*p* < 0.05), and from 1.13 to 1.78 (*p* < 0.01), respectively; however, “Perceived usefulness of e-learning” remained unchanged. The scores on clinical questions increased significantly from 2.08 to 2.92 (*p* < 0.01) (Fig. 3).

Correlations between the results of “Understanding how to use KM” and the scores of the clinical questions before and after the e-learning, respectively were, evaluated by the Spearman rank correlation coefficient. However, no correlation was found for each: before e-learning rs=-0.049 *p* > 0.05, after rs = 0.034 *p* > 0.05.

## Discussion

To solve the problem of the lack of qualified Kampo instructors and insufficient lecture time, we developed a new Kampo e-learning course to improve students’ and novices’ understanding of KM and evaluated its effectiveness. The e-learning course was assigned as homework in September 2022. Pre- and post-surveys and tests were conducted to determine its effectiveness. The students’ awareness of Kampo medicine and their scores on clinical questions significantly improved after the course, which indicated its efficacy and confirmed it objectively and subjectively.

We previously reported problems and challenges regarding standardizing Kampo education discovered by conducting surveys on Kampo education in all Japanese medical schools in 2011 and 2019 [6, 7]. In those reports, the proportions of “Curriculum standardization”, “Preparation of standard textbooks”, “Fostering instructors responsible for Kampo education”, and “Introduction of Kampo education into postgraduate clinical training” were 63%, 51%, 65%, and 33% in 2011 and 57%, 51%, 71%, and 35% in 2019, respectively. These results suggested that the situation of Kampo education in Japan had not improved even after the revision of the Japanese medical education model core curriculum in 2016, in which “Outlining the characteristics of KM: Adaptation and pharmacological effects of major Kampo formulas” was specified along with the recommendation of complementary medicine education in the medical school curricula by the Japan Accreditation Council for Medical Education (JACME) in 2016. We also reported the actual conditions of Kampo education in all the pharmacy schools in Japan after the enforcement of the national 2015 core curriculum [11]. In that report, “Contents establishment of the curriculum”, “Preparing standard textbooks”, and “Selecting adequate teachers for KM clinical education” were the problems revealed and the same as those in medical schools. To solve those problems, a standardized textbook of KM, “Essential Lecture on Kampo Medicine”, was published by the Japan Council for Kampo Medical Education (JCKME) in 2020 [12]. For the remaining “Curriculum standardization”, “Fostering instructors responsible for Kampo education”, and “Introduction of Kampo education into clinical training”, this new e-learning course described in the present study could easily be made available, solving the main problems, by incorporating it into the usual lecture series, thus becoming an active learning tool for students, as well as being used as a reference tool in clinical routines for physicians.

It is commonly known that students’ attention spans for lectures are at most 10–15 min [13, 14]; therefore, the lecture time for each e-learning lesson was limited to approximately 13 min concentrating on the key points. The e-learning course consists of 12 lessons. The lessons on the 10 essential Kampo formulas were chosen from recommendations by specialists and included Kampo theory, symptoms for diagnoses, clinical evidence, differential prescriptions, and case presentations. Thus, the objective is that the students and novices will be able to prescribe KM after finishing the e-learning course provided they receive their licenses. The other two lessons are “Good use practices and adverse reactions” and “Basic concepts” in which the different approaches for diseases between Western and KM, tips for enhancing the effect of KM, adverse effects to be kept in mind, herbs to be aware of, and basic Kampo theory are explained. By taking these lessons, students and novices will be able to use KM alone or along with Western medicine. For easy listening, the narrations of each lesson were recorded by a professional narrator. To improve the participants’ motivation, a certificate of completion is awarded at the end of the course and can be used for credit to improve test scores. The Japanese version of the e-learning course was used in the present study. We also made an English version with English narrations to inform people worldwide about KM. The English version has the same contents with a supplemental lesson called “What is Kampo?” which is not included in the Japanese version. The lesson “What is Kampo?” contains history, characteristics, and current status of KM. Differences between Kampo and Western medicine and comparisons of decoctions and extracts are also presented [15].

To evaluate this e-learning course, all the third-year medical students in Tokai University School of Medicine, who were generally novices in KM, were selected as participants in this required course. To improve the response rate, the students were informed before the course that points would be added to their regular test scores when they showed their certificate of completion. Consequently, all of the participants responded to all the questions. Due to these measures, selection bias was minimal.

After the e-learning course, the scores on “Interest in KM”, “Necessity for KM”, and “Understanding of KM” significantly improved, indicating that the e-learning course was evaluated favorably by the students. The improvement in the scores on the clinical questions also indicated its effectiveness in Kampo medical education. The students’ interest before the course was similar to our previous data which showed that interest in KM tended to be high even before studying it [16]. The students’ understanding of KM improved considerably after the e-learning course in which they participated individually without any outside help from teachers.

Ito, et al. reported developing a Kampo e-learning program and using it in a fourth-year flipped classroom at Keio University School of Medicine [17]. Although 35.2% of the students completed the program, the satisfaction rate and the comprehension level of the subject matter in the flipped classroom session were 86.4% and 79.6%, respectively. Furthermore, 80.7% thought that the flipped classroom should be used. The differences between our study and theirs were that the questionnaire response and course completion rates were not 100%, and they only conducted the questionnaire after the course completion. The completion rate was likely low because the class was held just before the CBT (Computer Based Testing) test. In our study, however, the e-learning course was assigned as homework, and the students were informed that points would be added to their test scores with proof of the course completion. Even in courses where attendance is merely recorded, the attendance rate would not reach 100%. This reward concept likely increased the students’ motivation to attend and complete the course. Furthermore, the issuance of certificates upon successful completion of all 10 clinical questions suggests that, at minimum, the content of the e-learning module could be retained as short-term memory. Ito, et al. Also asked the students more about their awareness of flipped classrooms than that of KM and e-learning, which was what we focused on. Furthermore, they did not evaluate the students’ knowledge acquisition as evidenced by studying using their e-learning course. Contrarily, we reported the objective and subjective efficacy and provided the confirming statistical analyses comparing data collected before and after the course. To our knowledge, this is the first report that confirms the effectiveness of Kampo e-learning by objective and subjective evaluation comparing data from before and after the course.

Noteworthy, Kainuma, et al. developed versatile and interactive model lessons to teach KM [18]. They evaluated the effectiveness and availability of Kampo specialists and non-specialists and reported that non-specialists were comparatively, relatively passive in using the model while discussing cases from a Kampo point of view. This was because non-specialists had few opportunities to discuss case reports from the perspective of therapy with KM. It can be deduced from their experience that if any Kampo education program is provided for classes given by non-specialists if the course is not self-contained, a preparatory instruction guide or pre-course seminar for the teachers must be provided. This is essential to develop proper Kampo educational materials. Our e-learning course, fortunately, contains lectures about Kampo formulas, related formulas, prescription guidelines, history, basic characteristics, and tests. Furthermore, students and novices can study using the course by themselves without any additional instruction from teachers. It is a completely self-contained learning course.

In the present study, the students’ perceived usefulness of e-learning did not change after they completed the course. The reason for this was unknown. However, we supposed that e-learning was not unfamiliar to most students who, due to the Covid-19 pandemic, had already experienced distance learning using their personal computers, tablets, and smartphones. In the present study population, there was a significantly high number of these students before the course began. Furthermore, the precise cause of the discrepancies between the results of “Understanding how to use KM” and the scores of the clinical questions before and after the e-learning, respectively, remains undetermined. However, it is presumed that nearly all of the students were novices in Kampo medicine. Through the e-learning process, they were introduced to and became familiar with Kampo medicine for the first time. Consequently, it seems that these scores independently improved.

The five limitations to this study were that the survey was conducted in a single facility and for only a 1-year period. A longitudinal study surveying multiple facilities is warranted and will generate more accurate results. Furthermore, this educational research design evaluated only short-term memory. The evaluation of long-term memory, which is a more significant educational goal, warrants further investigation. This investigation incorporated a reward system for participants. While not all students demonstrated a general inclination towards Kampo medicine, we postulated that the introduction of incentives would likely augment overall engagement and course completion rates, even among those initially disinterested in the subject matter. As a result, we opted to implement this strategic approach. Finally, in this study, the effect of e-learning was not compared with a control group. Typically, to assess the efficacy of an intervention, such a comparison is essential. For example, students are randomly assigned to either an e-learning group, a no-learning group or a traditional classroom group. However, it is often challenging for educators to differentiate educational opportunities for students. Consequently, this study evaluated the outcome before and after the implementation of a single e-learning course.

## Conclusions

We developed a new self-contained Kampo e-learning course for students and novices. After the participants completed the course, their awareness of KM increased and their scores on the clinical questions improved indicating that this new e-learning course in Kampo education is effective.

## Electronic supplementary material

Below is the link to the electronic supplementary material.

Supplementary Material 1

## Data Availability

The datasets used and analyzed during the present study are available from the corresponding author upon reasonable request.

## Abbreviations

KM: Kampo medicine
