# Supplementary material for: The effectiveness of a Kampo e-learning course incorporated into the medical education curriculum: a possible solution to instructor shortages and time constraints
Source: BMC Med Educ. 2025 Feb 24;25:299. doi: 10.1186/s12909-025-06874-9 (PMC11854378; doi:10.1186/s12909-025-06874-9)
Supplement: Supplementary file 1 — Supplementary Material 1 [file 12909_2025_6874_MOESM1_ESM.pptx]

## Slide 1
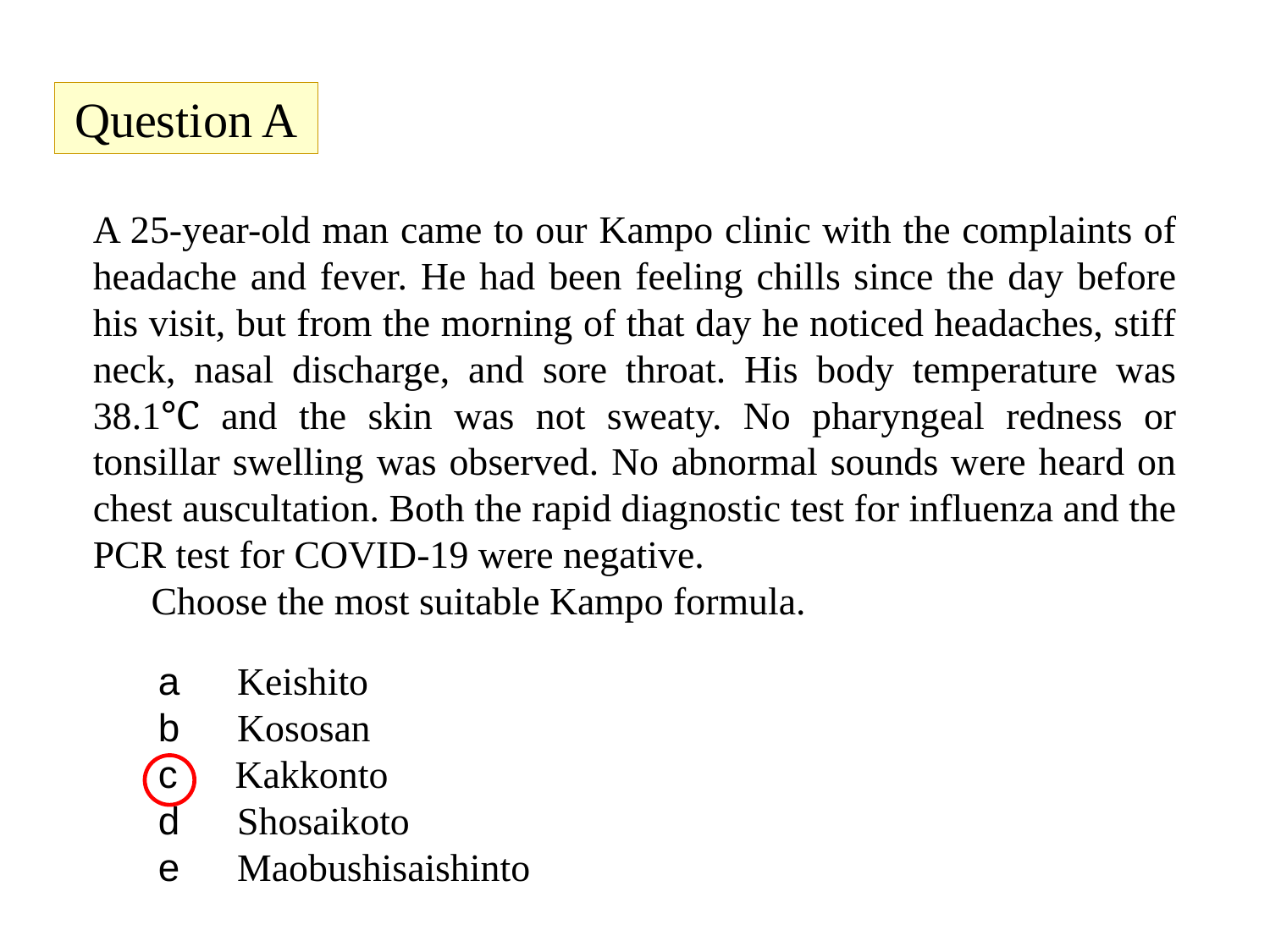

Question A
A 25-year-old man came to our Kampo clinic with the complaints of headache and fever. He had been feeling chills since the day before his visit, but from the morning of that day he noticed headaches, stiff neck, nasal discharge, and sore throat. His body temperature was 38.1℃ and the skin was not sweaty. No pharyngeal redness or tonsillar swelling was observed. No abnormal sounds were heard on chest auscultation. Both the rapid diagnostic test for influenza and the PCR test for COVID-19 were negative.
　 Choose the most suitable Kampo formula.
a　Keishito
b　Kososan
c　Kakkonto
d　Shosaikoto
e　Maobushisaishinto

## Slide 2
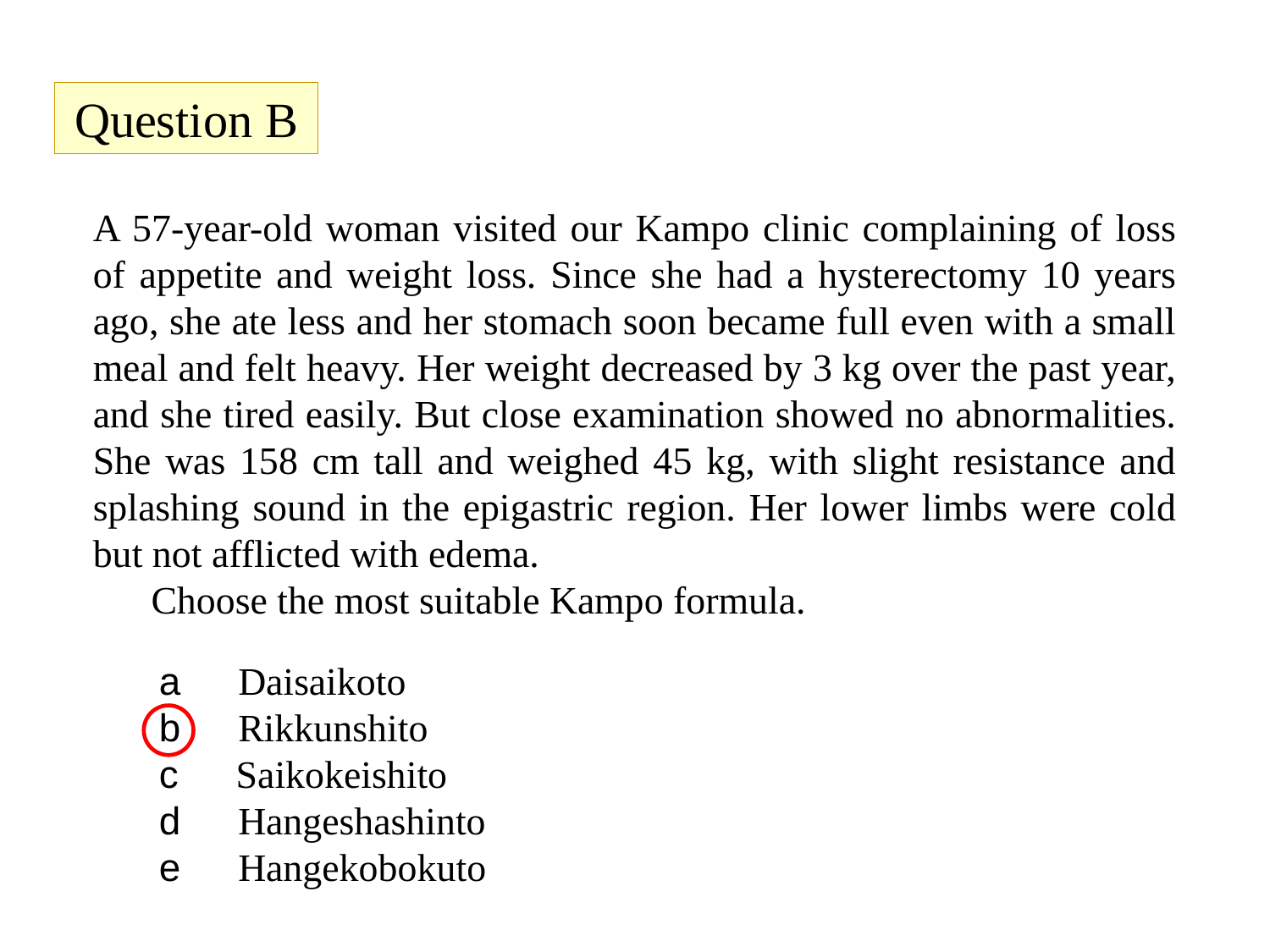

Question B
A 57-year-old woman visited our Kampo clinic complaining of loss of appetite and weight loss. Since she had a hysterectomy 10 years ago, she ate less and her stomach soon became full even with a small meal and felt heavy. Her weight decreased by 3 kg over the past year, and she tired easily. But close examination showed no abnormalities. She was 158 cm tall and weighed 45 kg, with slight resistance and splashing sound in the epigastric region. Her lower limbs were cold but not afflicted with edema.
　 Choose the most suitable Kampo formula.
a　Daisaikoto
b　Rikkunshito
c　Saikokeishito
d　Hangeshashinto
e　Hangekobokuto

## Slide 3
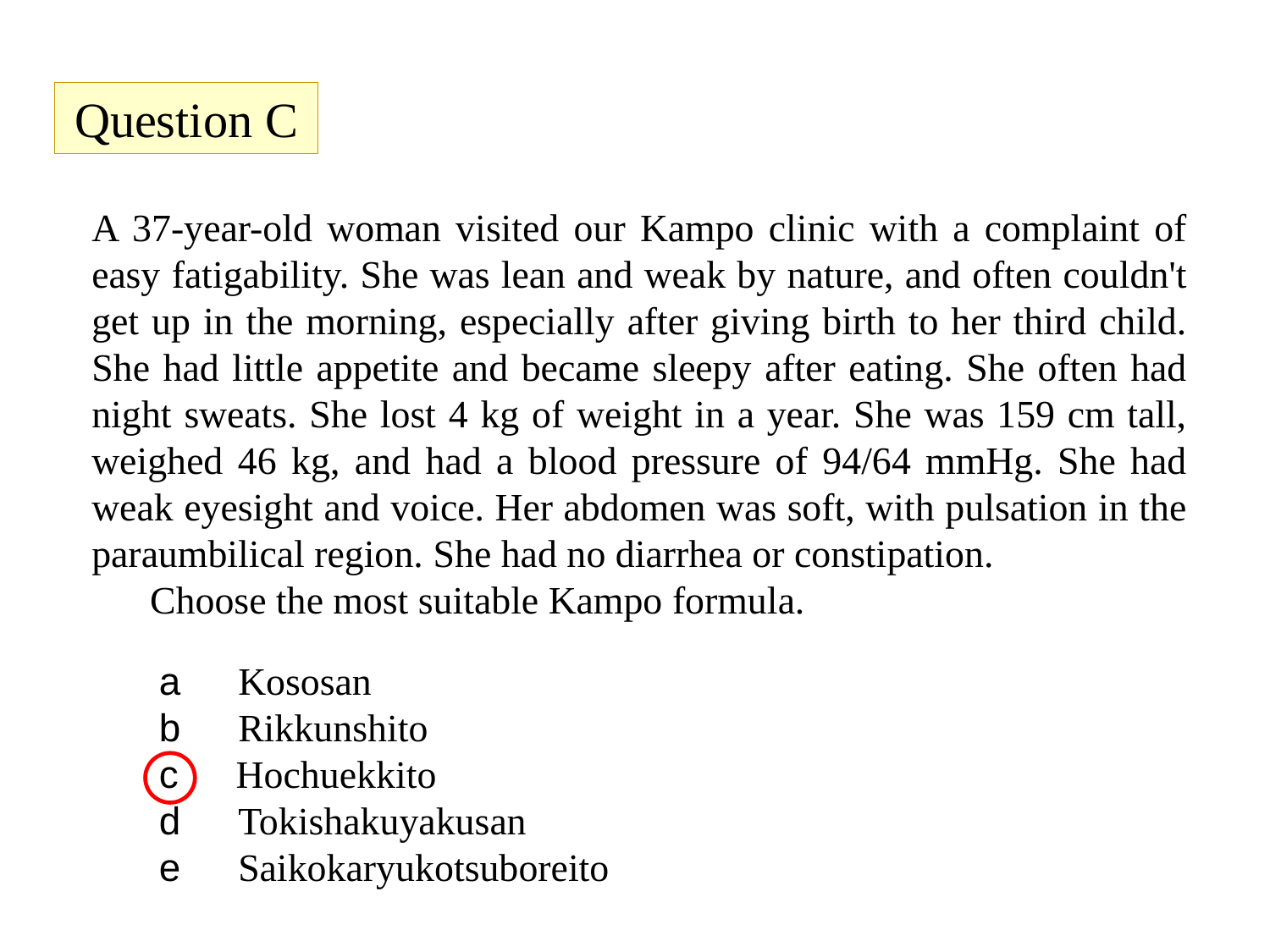

Question C
A 37-year-old woman visited our Kampo clinic with a complaint of easy fatigability. She was lean and weak by nature, and often couldn't get up in the morning, especially after giving birth to her third child. She had little appetite and became sleepy after eating. She often had night sweats. She lost 4 kg of weight in a year. She was 159 cm tall, weighed 46 kg, and had a blood pressure of 94/64 mmHg. She had weak eyesight and voice. Her abdomen was soft, with pulsation in the paraumbilical region. She had no diarrhea or constipation.
　 Choose the most suitable Kampo formula.
a　Kososan
b　Rikkunshito
c　Hochuekkito
d　Tokishakuyakusan
e　Saikokaryukotsuboreito

## Slide 4
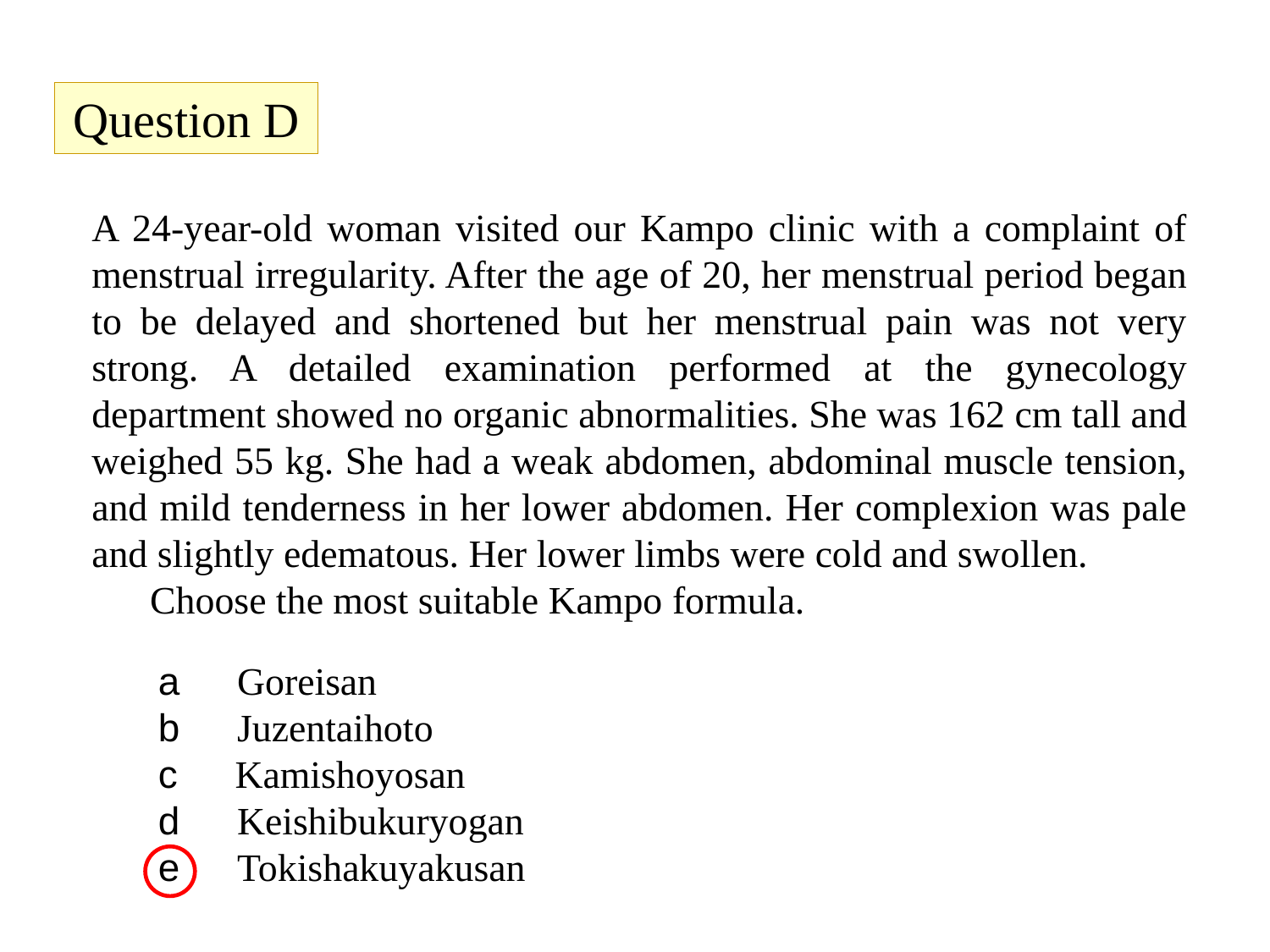

Question D
A 24-year-old woman visited our Kampo clinic with a complaint of menstrual irregularity. After the age of 20, her menstrual period began to be delayed and shortened but her menstrual pain was not very strong. A detailed examination performed at the gynecology department showed no organic abnormalities. She was 162 cm tall and weighed 55 kg. She had a weak abdomen, abdominal muscle tension, and mild tenderness in her lower abdomen. Her complexion was pale and slightly edematous. Her lower limbs were cold and swollen.
　 Choose the most suitable Kampo formula.
a　Goreisan
b　Juzentaihoto
c　Kamishoyosan
d　Keishibukuryogan
e　Tokishakuyakusan

## Slide 5
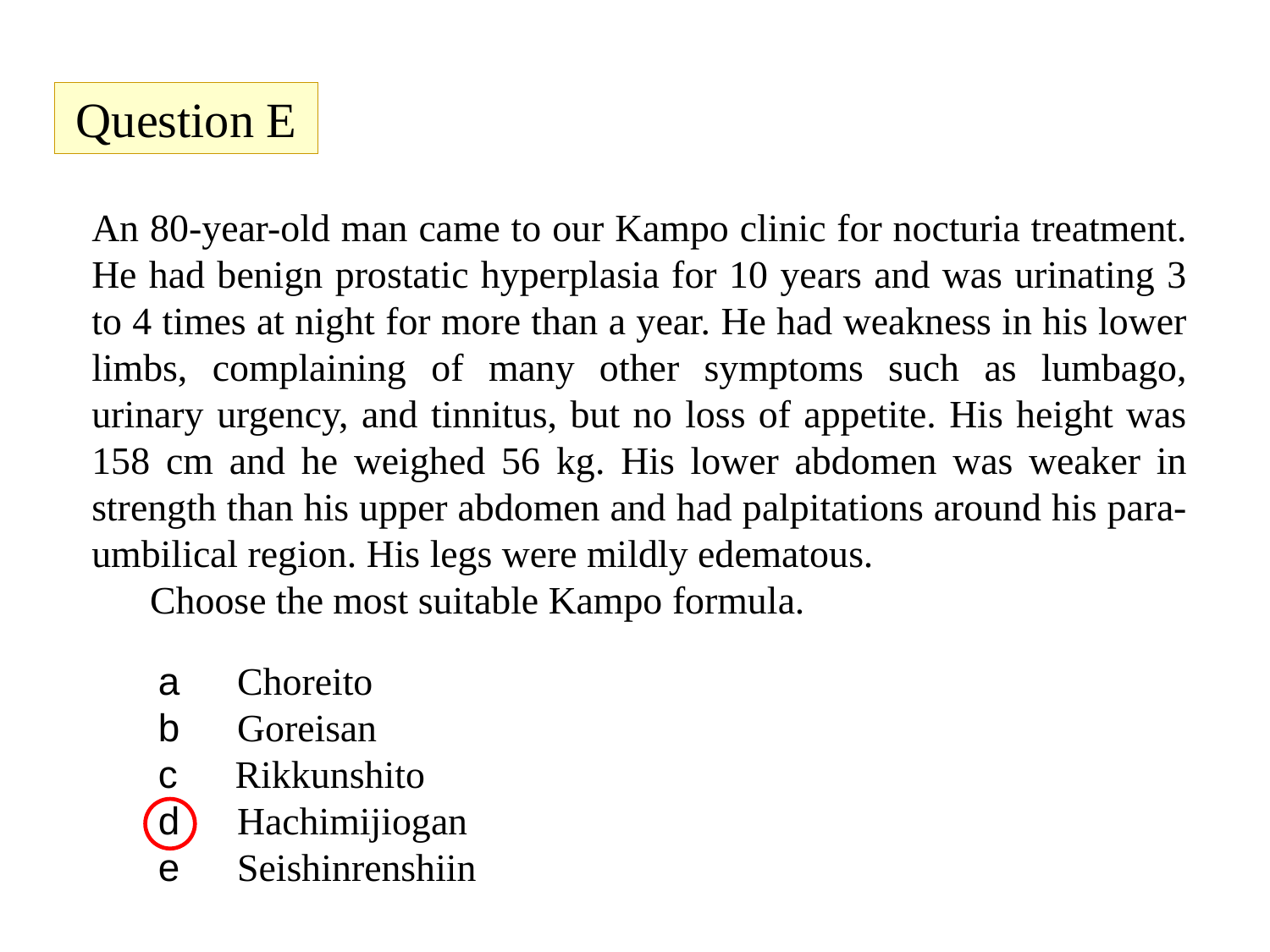

Question E
An 80-year-old man came to our Kampo clinic for nocturia treatment. He had benign prostatic hyperplasia for 10 years and was urinating 3 to 4 times at night for more than a year. He had weakness in his lower limbs, complaining of many other symptoms such as lumbago, urinary urgency, and tinnitus, but no loss of appetite. His height was 158 cm and he weighed 56 kg. His lower abdomen was weaker in strength than his upper abdomen and had palpitations around his para-umbilical region. His legs were mildly edematous.
　 Choose the most suitable Kampo formula.
a　Choreito
b　Goreisan
c　Rikkunshito
d　Hachimijiogan
e　Seishinrenshiin

## Slide 6
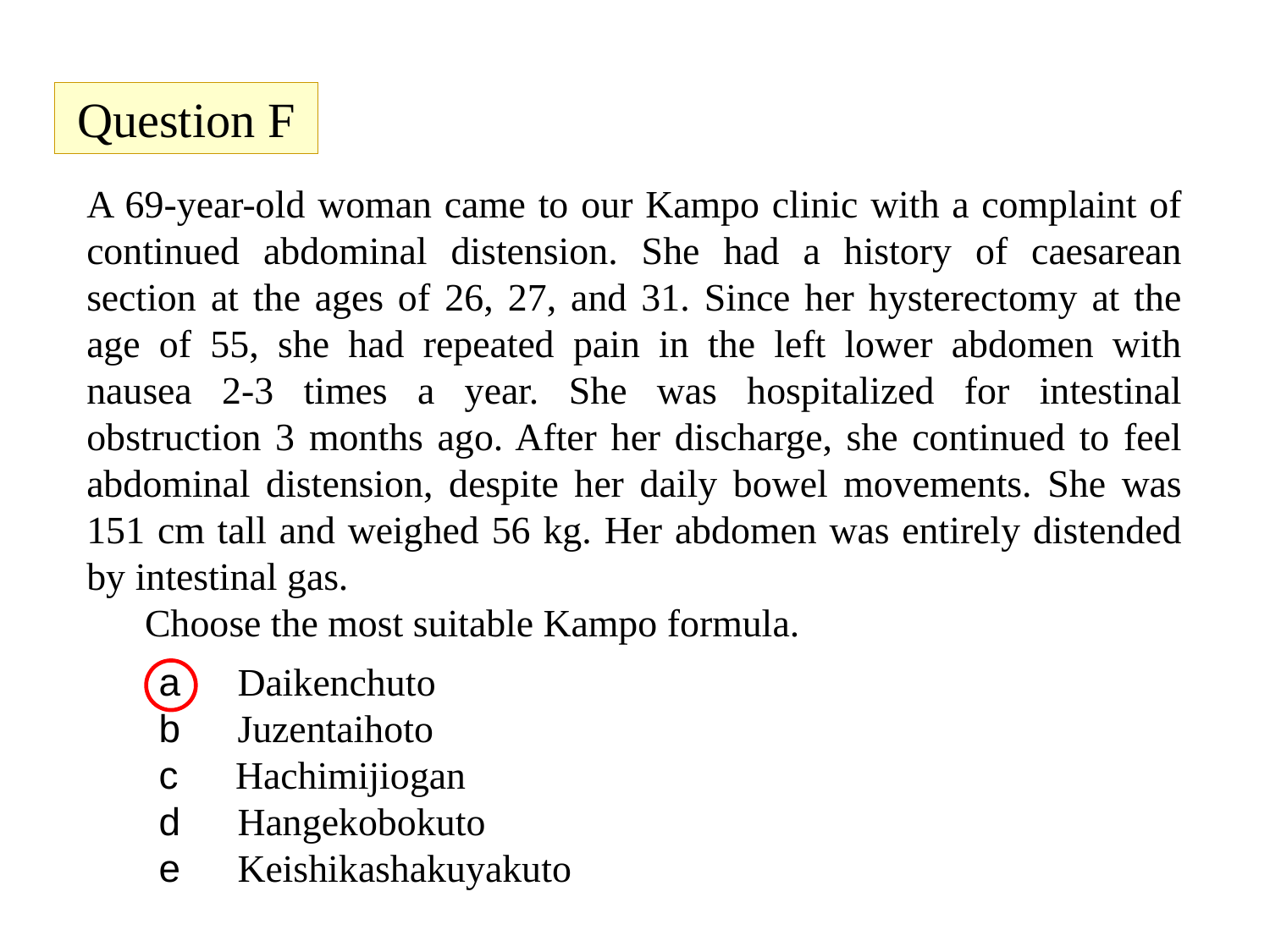

Question F
A 69-year-old woman came to our Kampo clinic with a complaint of continued abdominal distension. She had a history of caesarean section at the ages of 26, 27, and 31. Since her hysterectomy at the age of 55, she had repeated pain in the left lower abdomen with nausea 2-3 times a year. She was hospitalized for intestinal obstruction 3 months ago. After her discharge, she continued to feel abdominal distension, despite her daily bowel movements. She was 151 cm tall and weighed 56 kg. Her abdomen was entirely distended by intestinal gas.
　 Choose the most suitable Kampo formula.
a　Daikenchuto
b　Juzentaihoto
c　Hachimijiogan
d　Hangekobokuto
e　Keishikashakuyakuto

## Slide 7
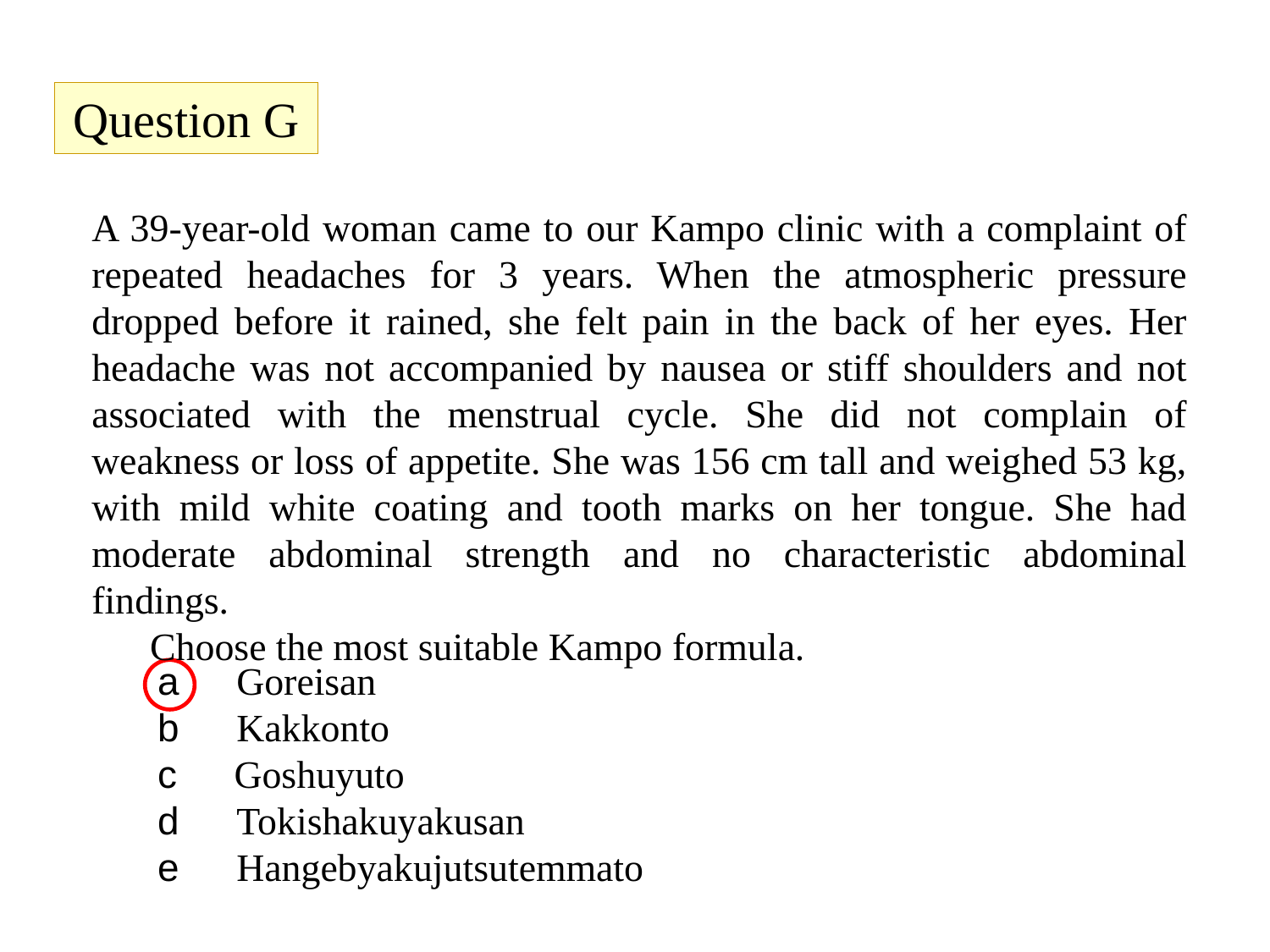

Question G
A 39-year-old woman came to our Kampo clinic with a complaint of repeated headaches for 3 years. When the atmospheric pressure dropped before it rained, she felt pain in the back of her eyes. Her headache was not accompanied by nausea or stiff shoulders and not associated with the menstrual cycle. She did not complain of weakness or loss of appetite. She was 156 cm tall and weighed 53 kg, with mild white coating and tooth marks on her tongue. She had moderate abdominal strength and no characteristic abdominal findings.
　 Choose the most suitable Kampo formula.
a　Goreisan
b　Kakkonto
c　Goshuyuto
d　Tokishakuyakusan
e　Hangebyakujutsutemmato

## Slide 8
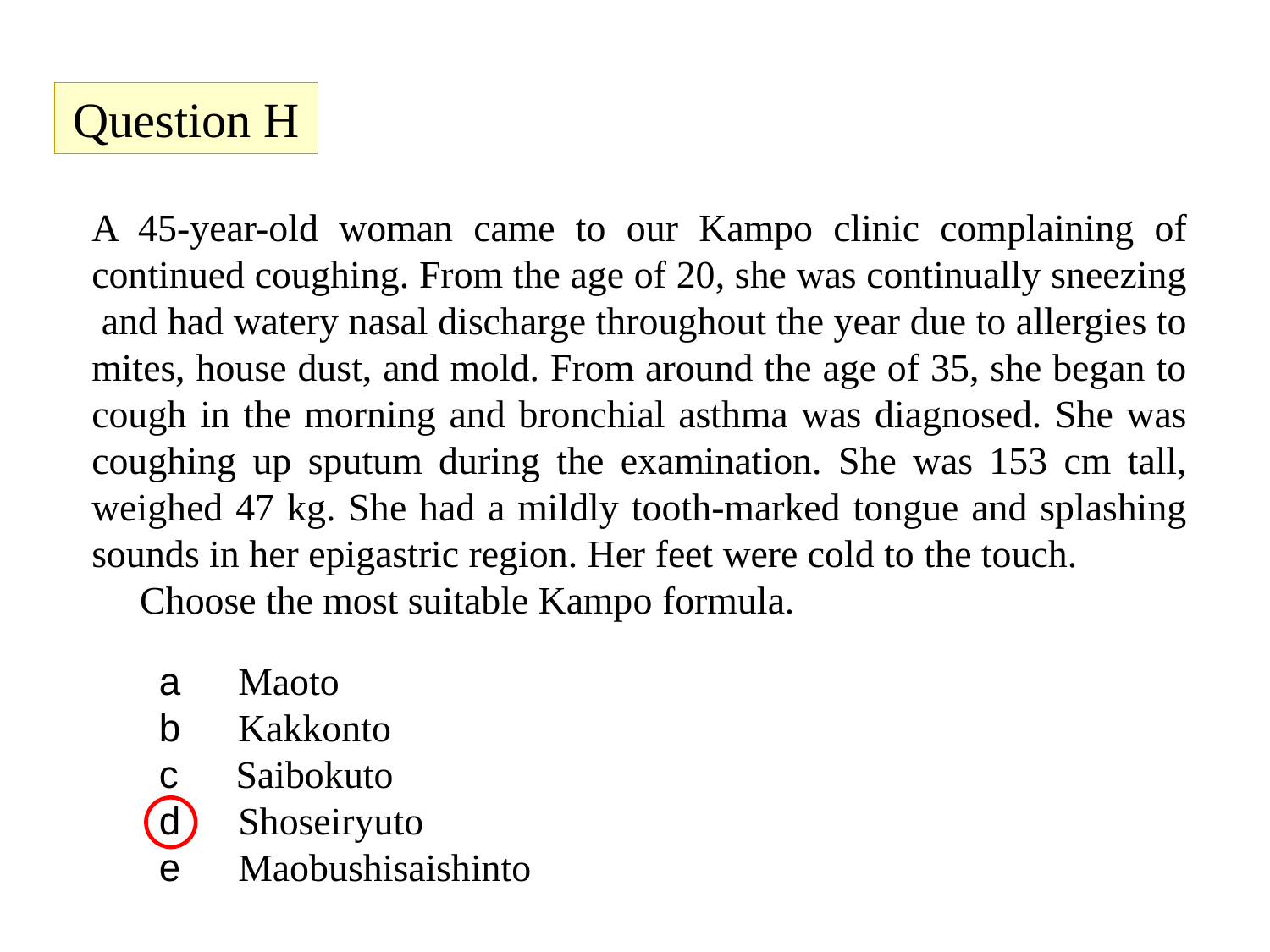

Question H
A 45-year-old woman came to our Kampo clinic complaining of continued coughing. From the age of 20, she was continually sneezing and had watery nasal discharge throughout the year due to allergies to mites, house dust, and mold. From around the age of 35, she began to cough in the morning and bronchial asthma was diagnosed. She was coughing up sputum during the examination. She was 153 cm tall, weighed 47 kg. She had a mildly tooth-marked tongue and splashing sounds in her epigastric region. Her feet were cold to the touch.
　Choose the most suitable Kampo formula.
a　Maoto
b　Kakkonto
c　Saibokuto
d　Shoseiryuto
e　Maobushisaishinto

## Slide 9
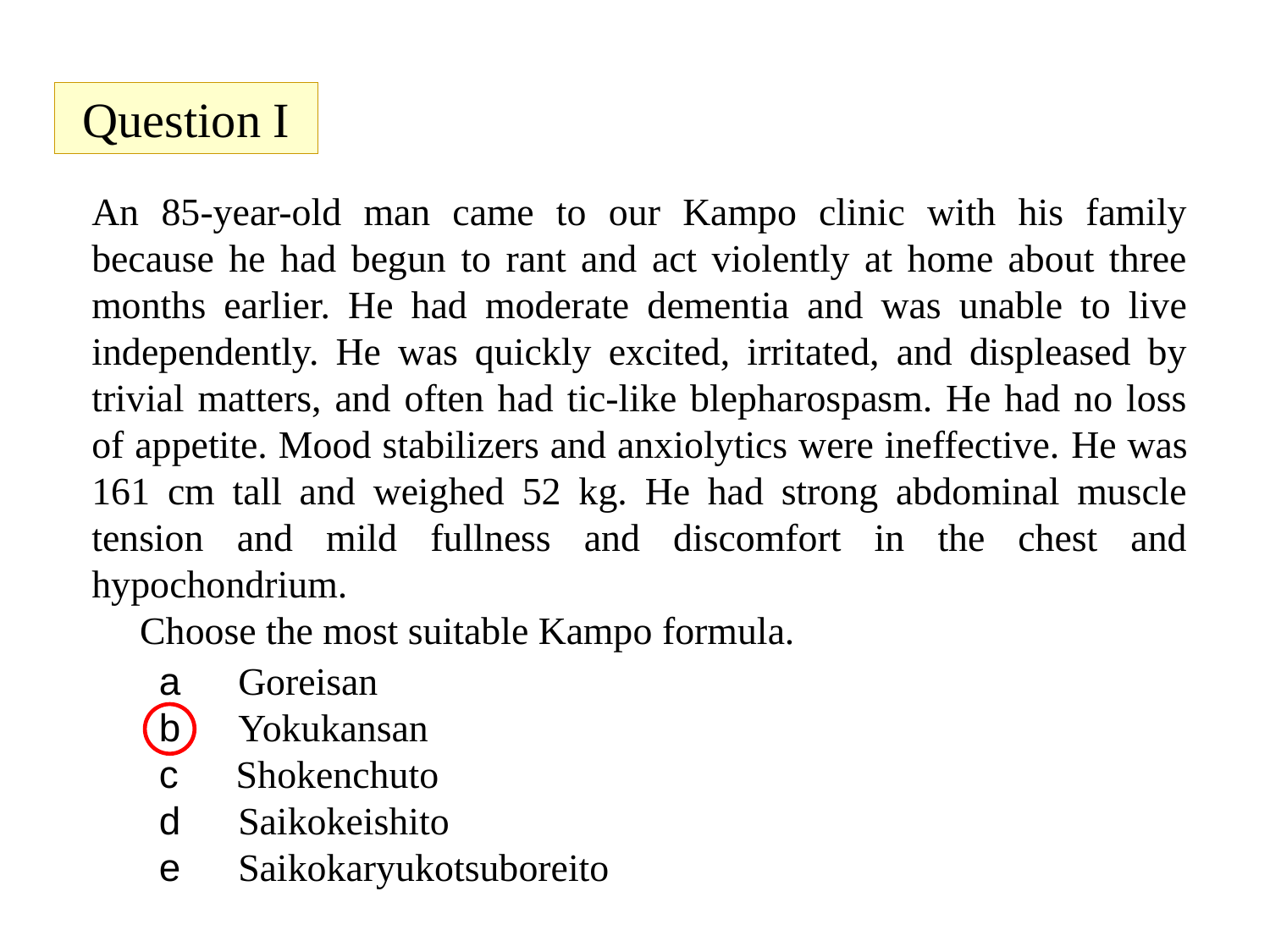

Question I
An 85-year-old man came to our Kampo clinic with his family because he had begun to rant and act violently at home about three months earlier. He had moderate dementia and was unable to live independently. He was quickly excited, irritated, and displeased by trivial matters, and often had tic-like blepharospasm. He had no loss of appetite. Mood stabilizers and anxiolytics were ineffective. He was 161 cm tall and weighed 52 kg. He had strong abdominal muscle tension and mild fullness and discomfort in the chest and hypochondrium.
　Choose the most suitable Kampo formula.
a　Goreisan
b　Yokukansan
c　Shokenchuto
d　Saikokeishito
e　Saikokaryukotsuboreito

## Slide 10
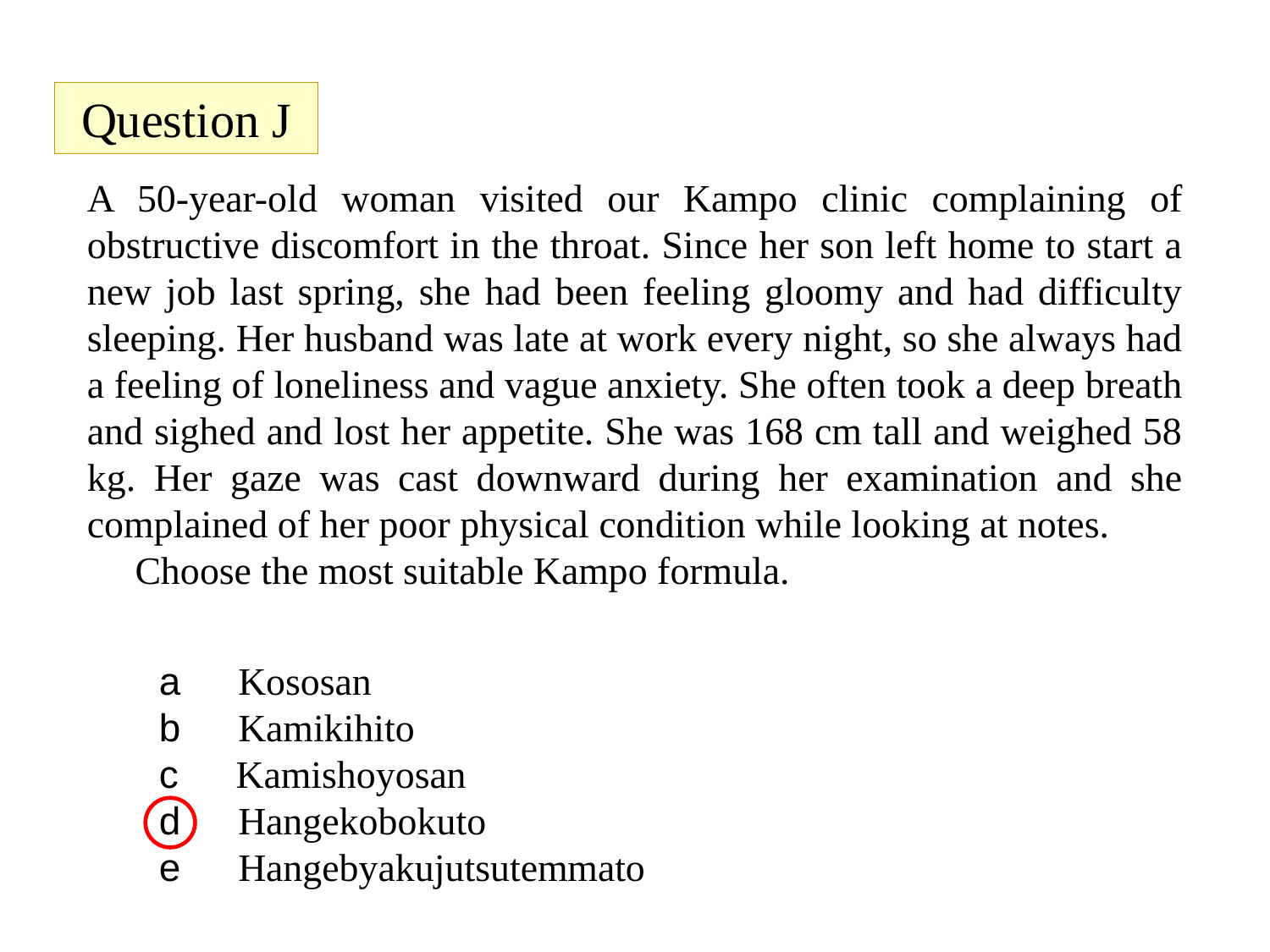

Question J
A 50-year-old woman visited our Kampo clinic complaining of obstructive discomfort in the throat. Since her son left home to start a new job last spring, she had been feeling gloomy and had difficulty sleeping. Her husband was late at work every night, so she always had a feeling of loneliness and vague anxiety. She often took a deep breath and sighed and lost her appetite. She was 168 cm tall and weighed 58 kg. Her gaze was cast downward during her examination and she complained of her poor physical condition while looking at notes.
　Choose the most suitable Kampo formula.
a　Kososan
b　Kamikihito
c　Kamishoyosan
d　Hangekobokuto
e　Hangebyakujutsutemmato
